# Silver Nanoparticles and Ionic Silver Separation Using a Cation-Exchange Resin. Variables Affecting Their Separation and Improvements of AgNP Characterization by SP-ICPMS

**DOI:** 10.3390/nano11102626

**Published:** 2021-10-06

**Authors:** Mònica Iglesias, Laura Torrent

**Affiliations:** 1 Department of Chemistry, University of Girona, C/M. Aurèlia Capmany, 69, 17003 Girona, Spain; 2 Bioenergy and Catalysis Laboratory (LBK), Energy and Environment Research Division (ENE), Paul Scherrer Institute (PSI), Forschungsstrasse 111, 5232 Villigen, Switzerland; laura.torrent@psi.ch

**Keywords:** silver nanoparticles, cation-exchange resin, environmental waters, single particle inductively coupled plasma mass spectrometry

## Abstract

Silver nanoparticles (AgNPs) are frequently found in everyday products and, as a consequence, their release into the environment cannot be avoided. Once in aquatic systems, AgNPs interact with natural constituents and undergo different transformation processes. Therefore, it is important to characterize and quantify AgNPs in environmental waters in order to understand their behavior, their transformation, and their associated toxicological risks. However, the coexistence of ionic silver (Ag^+^) with AgNPs in aquatic systems is one of the greatest challenges for the determination of nanosilver. Ion-exchange resins can be used to separate Ag^+^ from AgNPs, taking advantage of the different charges of the species. In this work, Dowex 50W-X8 was used to separate Ag^+^ and AgNPs in order to easily determine AgNP concentrations using inductively coupled plasma optical emission spectroscopy. The separation methodology was successfully applied to river water samples with different ratios of Ag^+^ and AgNPs. However, the methodology is not useful for wastewater samples. The described methodology also demonstrated an improvement in the determination of the particle size of AgNPs present in river waters by single particle inductively coupled plasma mass spectrometry when a significant amount of Ag^+^ is also present.

## 1. Introduction

The number of commercial products available on the market containing engineered nanoparticles (ENPs) is continuously growing. These nanomaterials are widely used in the industry because of their small size (<100 nm), which give them novel properties compared to their bulk counterparts. In our daily life, one of the common ENPs that can be found in everyday products are silver nanoparticles (AgNPs) because of their antibacterial properties [1,2]. According to *The Project of Emerging Nanotechnologies* and *The Nanodatabase*, AgNPs are used worldwide in textiles, household appliances, hygienic products, electronic devices, food and beverage packaging, medical appliances, and goods for children [3,4]. Because of their large production, the release of these nanoparticles into the environment during their manufacture, usage, and disposal is unavoidable [5]. Among the three environmental compartments (air, soil, and water), these emerging pollutants accumulate in different aquatic systems through water pollution remediation and industrial or wastewater treatment plant discharges. Another source of AgNP entry is through landfills, or through agricultural activities, such as the application of sewage sludge, or the usage of nanopesticides, as these particles can be leached from land and reach surface waters or groundwaters [6,7,8,9]. Through these routes of entry, nanosilver can be transported, both in their original form or after undergoing transformations [10,11]. In waters, AgNPs interact with natural constituents and also go through different transformation processes that may modify their physicochemical properties [2,6,12,13]. These phenomena alter the fate, transport, bioavailability, and toxicity of AgNPs. Dissolution is one of the key transformation processes that they can undergo in aquatic environments [2,12,14]. The resulting ionic silver (Ag^+^) from the metallic nanoparticles (NPs) dissolution is considered to be linked to the toxicity of AgNPs [5,12]. Therefore, concerns about the hazards that these emerging pollutants present to aquatic living organisms has increased. In fact, there are several studies that show that both AgNPs and Ag^+^ exert toxicity effects on algae (e.g., reduction of growth), plants (e.g., decrease of photosynthetic pigments), animals (e.g., lowering of locomotor mobility), and other living organisms [15,16,17,18,19,20]. For this reason, it is important to characterize and quantify AgNPs in environmental waters in order to understand their behavior, their transformation, and the associated toxicological risks. However, the coexistence of Ag^+^ with AgNPs in aquatic systems is one of the greatest challenges for the determination of nanosilver in this environmental compartment [21].

In the last few years, diverse analytical methodologies have been presented to overcome this analytical problem. Field flow fractionation (FFF) has been used, in combination with inductively coupled plasma mass spectrometry (ICPMS), for tracking the dissolution of AgNPs in laboratory, processed, and natural waters [22]. FFF has also been used, in combination with other analytical tools, for characterizing and quantifying both silver species in spiked surface waters [23]. The hyphenation of chromatographic methods with ICPMS has also been demonstrated to be effective for the separation and quantification of particulate and ionic silver, and for tracking their dissolution and size transformation [24,25]. However, one of the analytical methodologies that has been gaining in popularity over the years is single particle inductively coupled plasma mass spectrometry (SP-ICPMS), which allows the identification, characterization, and determination of the mass and particle number concentration of AgNPs at low concentrations (ng·L^−1^ level) [21]. In fact, SP-ICPMS has been employed to obtain quantitative and qualitative information on AgNPs present in spiked or non-spiked lake waters [26,27,28,29], river waters [28,30,31], tap waters [30,32], seawaters [33,34,35,36], and wastewaters [36,37,38]. Despite the advantages of these sophisticated methods, they also present some drawbacks. For example, low recoveries in FFF because of the interaction of the NPs with the working membranes [39]. In the case of SP-ICPMS, the presence of high amounts of ionic species of the same element as the analyte can inhibit the distinction of AgNPs and Ag^+^, and increase the particle size limit of detection [40]. For this reason, simpler and less expensive analytical tools for detecting, extracting, and/or preconcentrating AgNPs have been developed. Alternatives, such as stripping voltammetry or cyclic voltammetry, have been used to detect nanosilver in the presence of Ag^+^ in real waters [41,42,43]. Sample pretreatment methods have also been employed in combination with the aforementioned analytical techniques. A simple approach, such as ultrafiltration, has been used in combination with ICPMS in order to determine the Ag^+^ in aqueous samples [27]. Another methodology that has been extensively used, in combination with diverse spectrometric techniques, is cloud point extraction to preconcentrate and separate AgNPs from Ag^+^ in different spiked and non-spiked environmental waters, without changing their characteristics [44,45,46,47,48,49,50,51,52,53,54,55]. However, this methodology also presents some drawbacks. For instance, the recoveries of AgNPs are neither easily reproducible nor low because of some critical steps, such as the pH adjustment. To a lesser extent, magnetic solid phase extraction has also been used, in combination with SP-ICPMS, to selectively separate both silver chemical forms (nanoforms and ionic forms) [56,57]. Another possible option for separating the AgNPs and Ag^+^ is the use of solid phase extraction, taking into account the ability to separate the different charged species. For example, an anionic-exchange resin was employed to extract different AgNPs present in wastewaters by modifying the surface with mercaptosuccinic acid for their adsorption into the resin. The extracted NPs were analyzed with graphite furnace atomic absorption spectrometry [58]. Apart from anionic-exchange resin, a method with cation-exchange resin has been developed for removing the Ag^+^ present in aqueous samples before AgNP characterization by SP-ICPMS analysis [59]. To our knowledge, this analytical methodology has been applied to wastewaters [59], soil extracts [60], and commercial suspensions [61]. The purpose of this work is to extend the application of this method to other natural waters. With this aim, the cation-exchange procedure was optimized, and the influence of some components present in natural waters on the extraction was also evaluated in order to have acceptable recoveries. In addition, two different setups (batch and column) were studied for performing the solid phase extraction. Finally, the pretreatment procedure was applied to two river waters and wastewater samples in combination with inductively coupled plasma optical emission spectroscopy (ICP-OES). One of the river waters was also analyzed in combination with SP-ICPMS in order to improve the characterization of the extracted AgNPs.

## 2. Materials and Methods

### 2.1. Chemicals, Materials, and Apparatus

Commercial polyvinylpyrrolidone-coated silver nanoparticles (PVP-AgNPs) of 75 nm, and 60 nm of citrate-stabilized bare silver nanoparticles were acquired from Nanocomposix (St. Louis, MO, USA). The zeta potentials of these nanoparticles are −37 and −54 mV, respectively. PVP-AgNPs were employed to prepare standards for the method development and the SP-ICPMS analysis. An ionic silver stock solution (1000 ± 2 mg·L^−1^), purchased from Merck (Darmstadt, Germany), was used to prepare the calibration standards for ICP-OES analysis, and to prepare spiked samples for the method development. Moreover, mixtures of both aforementioned AgNPs (citrate and PVP) with Ag^+^ were prepared for the method evaluation. 

To separate AgNPs from Ag^+^, Dowex 50W-X8 cation-exchange resin was used (Sigma-Aldrich, Darmstadt, Germany).

Analytical grade hiperpur-quality nitric acid (HNO_3_ 69%, Panreac, Barcelona, Spain) and sodium hydroxide (NaOH, Fisher Scientific, EUA, Waltham, MA, USA) were used for resin conditioning. Ultrapure water from a Milli-Q purification system (Millipore Corp., Bedford, MA, USA) was utilized to dilute standards, reagents, and samples. Humic acid (Sigma-Aldrich, Darmstadt, Germany) and calcium (Ca^2+^)stock solution (1000 ± 2 mg·L^−1^, Merck, Germany) were used to study the experimental conditions.

River water samples were obtained from the Osor River (Anglès and Osor, Girona, Spain) at two different points. The wastewater sample was collected from the wastewater treatment plant of Girona (Can Dura, Girona, Spain). The main characteristics of the waters are listed in Table 1.

Batch separation experiments were performed using a rotary mixer Dinko (Barcelona, Spain). An ultrasonic system, J.P. Selecta (Barcelona, Spain), was used to break down the possible agglomerated AgNPs. A P-Selecta oven was used to dry the resin before the ion-exchange procedure.

### 2.2. Instrumentation

Total Ag concentrations were determined using an Agilent Technologies Vertical Dual View 5100 ICP-OES (Tokyo, Japan). SP-ICPMS was performed with a quadrupole-based Agilent Technologies model 7500c ICPMS (Tokyo, Japan) equipped with an octapole reaction system (ORS). SP-ICPMS data treatment was carried out using an in-lab Microsoft Excel spreadsheet. The separation of the signal corresponding to the AgNPs from the Ag^+^ was performed by employing the iterative algorithm based on 5 times the standard deviation, plus the mean of all data points, obtained from the analysis [62]. In order to transfer the particle intensity to particle mass, the nebulization efficiency was calculated using the particle frequency method [63]. This method uses the number of particle events detected during a fixed acquisition time, the particle number concentration, and the sample flow rate to calculate the nebulization efficiency. The nebulization efficiencies obtained using the in-lab Microsoft Excel spreadsheet were comparable to the ones obtained using the RIKILT spreadsheet [64], with the advantage of not needing to use silver standards. The instrumental characteristics and measurement conditions of the different equipment used in this study are listed in Table 2. In addition, an ANOVA was performed to determine if there were statistical differences between the results obtained.

### 2.3. Separation Procedure

In order to carry out the separation of AgNPs from Ag^+^ in water samples, a simple ion-exchange methodology was used. Prior to its use, the cation-exchange resin was conditioned using 1.5 M HNO_3_. The resin can be used in this acid form or can be converted to its sodium form with 0.1 M NaOH solution. The steps followed in this study were obtained from [61].

- Batch Studies

In order to study the variables affecting the separation of AgNPs and Ag^+^, batch sorption studies were carried out. The required quantity of Dowex 50W-X8 resin was conditioned. Afterwards, 10 mL of the silver solution containing either AgNPs or Ag^+^, or mixtures of them, were added. This solution was rotated for 60 min and then the resin was left to settle down. The supernatant was extracted, and the silver concentration was determined in this supernatant solution by ICP-OES. When two extraction procedures were used, the supernatant was again put in contact with a freshly conditioned amount of resin, and the mixture was rotated once again for 60 min. The supernatant was again separated from the resin, and the silver concentration was determined. The fraction of silver that remains in the supernatant, named the “Remaining silver fraction (%)”, was calculated according to the following equation:

Remaining silver fraction (%) = [Ag]_f_/[Ag]_i_ × 100 (1)

where [Ag]_f_ is the remaining silver concentration after the adsorption process, and [Ag]_i_ is the initial silver concentration.

- Column Studies

The separation of AgNPs and Ag^+^ was also studied under column conditions. A small amount of filter paper was placed at the bottom of a cut polypropylene Pasteur pipette, and 0.5 g of the resin was placed inside. The resin was then conditioned with 1.5 M HNO_3_ and transformed to its sodium form using 0.1 M NaOH solution. Silver solutions, either AgNPs or Ag^+^, were passed through the resin bed, and the eluent of the column was collected in polypropylene tubes for analysis. The flow rates used in these experiments were from 1.2–1.4 mL·min^−1^.

## 3. Results and Discussion

### 3.1. Effect of the Material Container without the Presence of Resin

**Figure 1:**
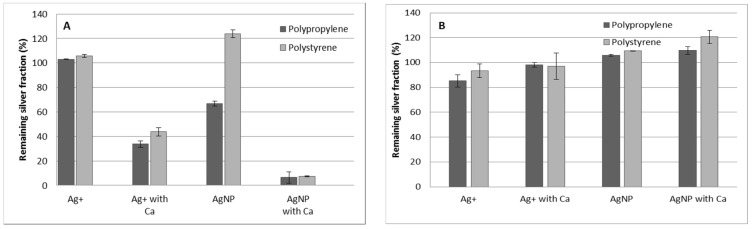
Remaining silver fraction using different tube materials without the presence of resin. (**A**) In the absence of humic acids. (**B**) In the presence of humic acids. Error bars represent standard deviation n = 2.

Prior to the study of the separation, the stability of the silver solutions, both Ag^+^ and AgNPs (75 nm PVP-AgNPs), were tested under different conditions and using containers of different materials (polypropylene and polystyrene). For this purpose, solutions of 100 μg·kg^−^^1^ and different matrix compositions were rotated for 60 min using the same conditions as the adsorption study, but without the presence of the resin. The results of these tests can be seen in Figure 1A. The presence of Ca^2+^ (40 mg·kg^−^^1^) in the silver solutions dramatically affected the stability of the analytes, resulting in losses of more than 55% for both tested materials. The effect was even more prominent in AgNP suspensions, which showed remaining silver fractions below 10%.

This behavior can be explained by the adsorption process to the tube walls that is only effective in the presence of Ca^2+^. To verify this adsorption process, the tubes were emptied, rinsed with Milli-Q water and, afterwards, 10 mL of 5 M HNO_3_ was added. Then, the tubes were rotated for 24 h and, finally, the amount of silver was determined in the acid solution. 80% of the silver, which previously dissipated, was recovered in the acidic solution, confirming the retention of silver in the tube walls. The presence of Ca^2+^ in the AgNP solutions has been previously related to the agglomeration effect [65]. The explanation of both processes (agglomeration and adsorption) may be due to similar mechanisms, and possibly related to the surface charge of the nanoparticles.

In order to solve this stability problem, the presence of humic acid (25 mg·kg^−^^1^) in the silver solution was tested. Previous studies [65,66] indicate that humic acids stabilize AgNPs and hamper their agglomeration. The results obtained can be seen in Figure 1B. Good stabilities of both Ag^+^ and AgNPs were obtained using humic acids, either with or without the presence of Ca^2+^ in the solutions.

An ANOVA test indicated that there were no significant differences between the container materials tested when humic acids were present (*p*-values = 0.26, 0.88 and 0.13, respectively), except in the case of AgNPs without calcium (*p*-value = 0.02). This could possibly be because of the unusually low standard deviations obtained in this experiment. Therefore, taking into account these results, polystyrene tubes were used in the rest of the work.

### 3.2. Resin Conditioning and Contact Time

**Figure 2:**
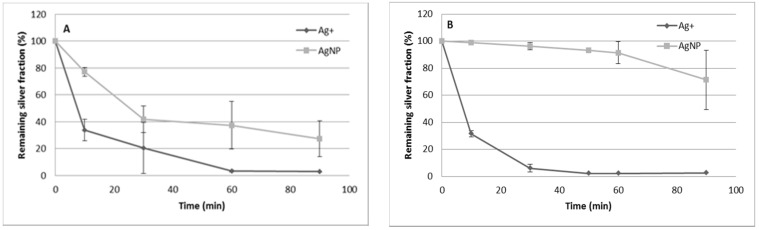
Remaining silver fraction vs. contact time. (**A**) Resin in acid form. (**B**) Resin in sodium form. Error bars represent standard deviation n = 2. Initial silver concentration is 57 μg·kg^−1^ for Ag^+^, and 85 μg·kg^−1^ for AgNPs.

As previously mentioned, the resin can be conditioned in acid or sodium form. The results of both types of conditioning for Ag^+^ and AgNPs adsorption in relation to time were studied, and the results are shown in Figure 2. These experiments were done using 10 mg of the Dowex 50W-X8 resin. From this figure, the better behavior of the sodium form can clearly be seen, allowing the separation of both silver forms. These results are in agreement with those present in the literature that use the present resin in sodium form to carry out silver ion separation from AgNPs [60,61]. Moreover, as can be observed in Figure 2, from 30 to 60 min is the contact time that allows the better separation of both forms.

The adsorption procedure was carried out using different silver concentrations with both ionic silver and AgNPs, and the results obtained were very similar for concentrations ranging from 20 to 200 µg·Kg^−^^1^ (data not shown). Therefore, the separation methodology can be applied in a broad range of silver concentrations.

### 3.3. Effect of Calcium and Humic Acids in the Separation

**Figure 3:**
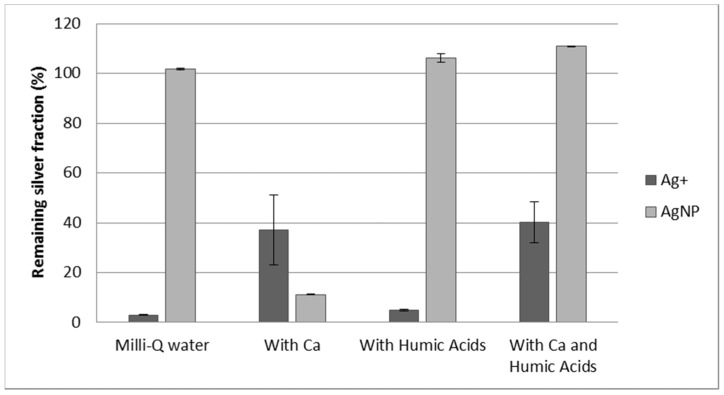
Remaining silver fraction (Ag^+^ or AgNPs) after 60 min of contact with 10 mg of resin in Na^+^ form. Error bars represent standard deviation n = 2. Initial silver concentration is 100 μg·kg^−^^1^ for both Ag^+^ and AgNPs.

The separation of Ag^+^ and AgNPs in water samples can be affected by the presence of interfering substances in the matrix. The presence of cations, mainly Ca^2+^, in freshwater samples, can hamper the adsorption of Ag^+^ by Dowex 50W-X8 resin because of a competing behavior. Therefore, the effect of the presence of Ca^2+^ in water samples in the recovery of both silver forms was studied. However, the instability of the solutions without humic acids, as previously observed, has to be taken into account. In Figure 3, the recoveries of silver (in the form of Ag^+^ or AgNPs) under different conditions using Dowex 50W-X8 is shown. Although the recoveries of silver nanoparticles are very low when Ca^2+^ is present, this behavior was also observed without resin in the tube (see Figure 1A). Therefore, the addition of humic acids to stabilize the AgNPs in the presence of Ca^2+^ becomes mandatory. As can be observed, the presence of humic acids, without calcium, does not affect the adsorption behavior of the silver species, indicating that it can be used to stabilize the solutions in the presence of calcium without any additional effect. The addition of both humic acids and Ca^2+^ to the silver solutions shows good stability of AgNPs. However, Ag^+^ adsorption is still worse than that observed in the absence of calcium. This is due to calcium cations competing for the resin. Therefore, although the separation is still feasible, the conditions must be improved in order to remove Ag^+^ more efficiently.

### 3.4. Effect of Resin Quantity, Double Extraction Procedure, and Reuse of the Resin

**Figure 4:**
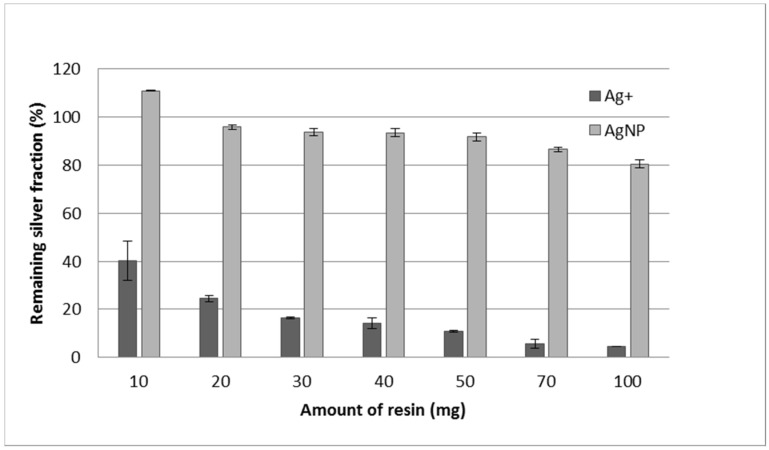
Remaining silver (Ag^+^ or AgNPs) after 60 min of contact with the resin in sodium form using different amounts. Error bars represent standard deviation n = 2. Initial silver concentration is 100 μg·kg^−^^1^ for both Ag^+^ and AgNPs.

To improve the separation of Ag^+^ and AgNPs, different amounts of resin were tested. As can be observed in Figure 4, the more the amount of resin, the less the amount of Ag^+^ remaining in the solution. However, for quantities of resin greater than 50 mg, the amount of AgNPs slightly decreases. Although, under these conditions, the separation is near 90%, we tried to improve it using a double extraction. The supernatant solution of the first extraction step was put in contact with a freshly conditioned amount of resin (50 mg again). After this second extraction process, the amount of Ag^+^ in the supernatant was 3.0% (S.D. 0.2), and the recovery of AgNPs was 82.4% (S.D. 1.3). Therefore, the separation is clearly improved by using two extraction processes.

**Figure 5:**
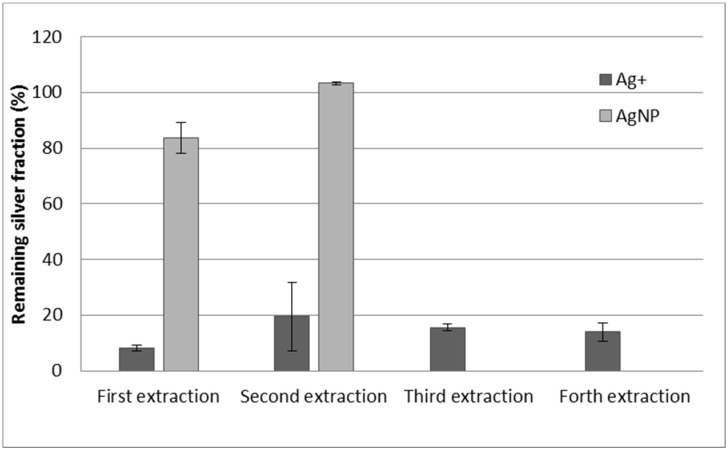
Reuse of the resin after 4 adsorption cycles. Error bars represent standard deviation n = 2. Initial silver concentration is 100 μg·kg^−^^1^ for both Ag^+^ and AgNPs.

The reuse of the resin was also studied. For this experiment, the resin used for the separation steps (first or second) was reconditioned in sodium form, following the same procedure used for conditioning the resin, and reused. As can be observed in Figure 5, the adsorption of silver ions is still very high using the reused resin, and the adsorption of AgNPs is almost negligible. The reuse of the resin was carried out four times for Ag^+^ and two times for AgNPs (after conditioning each time), and the results were very similar in all the cases, indicating the suitability of the reuse of the resin. Moreover, an ANOVA test was carried out and there were not significant differences (*p* > 0.05) in the remaining values of ionic silver between extraction cycles.

### 3.5. Analysis of Mixtures of Ag^+^/AgNPs and Application to River Water and Wastewater Samples

**Figure 6:**
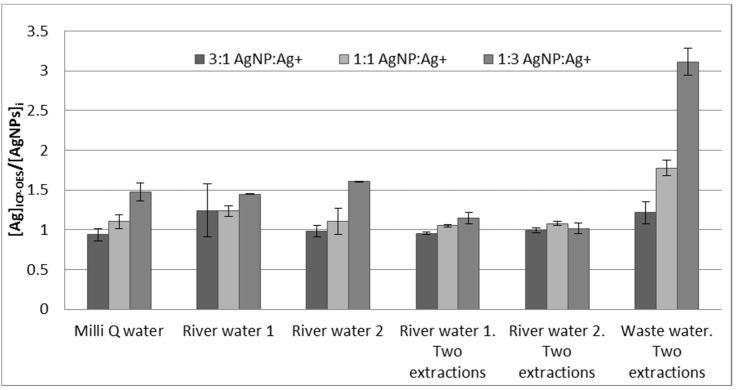
Total silver concentration measured by ICP-OES divided by AgNP initial concentration from mixtures of Ag^+^ and AgNPs in different types of water samples using single and double extraction procedures. Error bars represent standard deviation, n = 2. Total initial silver concentration (Ag^+^ + AgNPs) is 40 μg·kg^−^^1^ for all mixtures of Ag^+^ and AgNPs.

The effectiveness of the ion-exchange methodology for separating the AgNPs from Ag^+^ was tested using ICP-OES. The resulting aqueous extracts of the Ag^+^ and AgNPs mixtures at different proportions of different spiked water samples were analyzed. Due to the fact that we expected to find only AgNPs in the extracts, the results were calculated as the total silver concentration measured by ICP-OES in the aqueous extracts divided by the initial AgNP concentration. Therefore, when the separation works properly, we expect to obtain values close to 1. The results obtained from using either one or two extraction steps can be seen in Figure 6. As can be observed, for up to a 1:1 ratio, the measured concentrations of silver by ICP-OES were similar between the one and two extraction procedures. However, when the amount of Ag^+^ is higher, a double extraction procedure is necessary so as not to overestimate the amount of AgNPs. This behavior is observed with spiked Milli-Q and river water samples. In all these samples, humic acids have been added in order to stabilize the AgNPs. Note that neither the addition of humic acids (pH = 8.36), nor the extraction process (pH = 8.42), remarkably changes the pH of the tested River Water 1 (initial pH = 8.31).

The system was also applied to wastewater. However, the adsorption of silver cations in this type of water was very low. Even with 100 mg of resin and the application of a double extraction, the adsorption of silver ions only reached 25%. A possible explanation of this phenomenon is that the ionic strength of these waters was very high, and the presence of competing cations present in the sample was much higher, hampering the adsorption of silver ions. Therefore, additional studies of this system are needed to improve and achieve the separation of AgNPs and Ag^+^ in different types of environmental waters.

### 3.6. Separation of Ag^+^ and AgNPs Using Column Experiments

**Figure 7:**
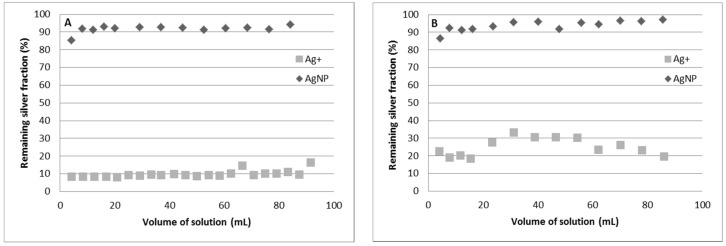
Remaining silver fraction (Ag^+^ or AgNPs) in column experiments using River Water 2 as matrix. (**A**) New resin. (**B**) Reconditioned resin. Silver concentration: 100 µg·kg^−^^1^; Flow rate: 1.3 mL·min^−^^1^; Amount of resin: 0.5 g.

The separation of both silver forms (Ag^+^ and AgNPs) was also studied under column experiments. In these experiments, 100 mL of 100 µg·Kg^−^^1^ silver solutions were passed through the column separately. As can be observed, the separation of both silver species is almost complete (higher than 90%) when a freshly conditioned resin is used. However, the reuse of the column, after reconditioning, shows a lower retention of silver ions and, as a result, a lower separation (Figure 7). Therefore, the column procedure can be used to separate Ag^+^ and AgNPs, but an improvement in the conditioning of the resin must be carried out in order to be able to reuse the resin.

### 3.7. Separation of Ag^+^ from AgNPs Previous to SP-ICPMS Analysis for a Better Quantification and Characterization of AgNPs

**Figure 8:**
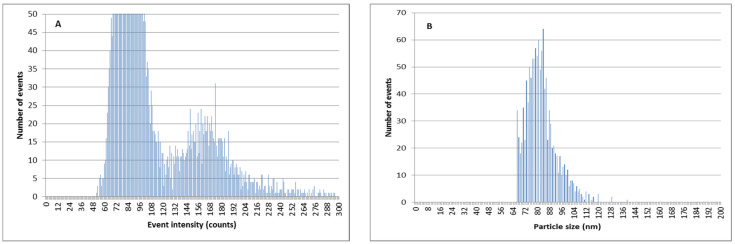
SP-ICPMS histograms of a mixture of Ag^+^ and PVP-AgNPs of 75 nm 5:1. (**A**) Signal distribution. (**B**) Particle size distribution.

**Figure 9:**
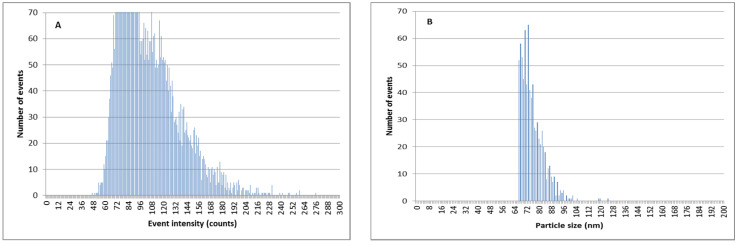
SP-ICPMS histograms of a mixture of Ag^+^ and citrate-stabilized AgNPs of 60 nm 5:1. (**A**) Signal distribution. (**B**) Particle size distribution.

The ion-exchange methodology can also be used in order to decrease the amount of Ag^+^ in AgNP suspensions and to improve the determination of AgNP size and quantity by SP-ICPMS. As can be observed in Figure 8, when silver nanoparticles are mixed with a relevant amount of ionic silver in river water samples, the discrimination between the signal coming from ionic silver, and that coming from AgNPs, is difficult. This led to poor results for particle size distributions. This effect is more pronounced for smaller silver nanoparticles of 60 nm (Figure 9).

**Figure 10:**
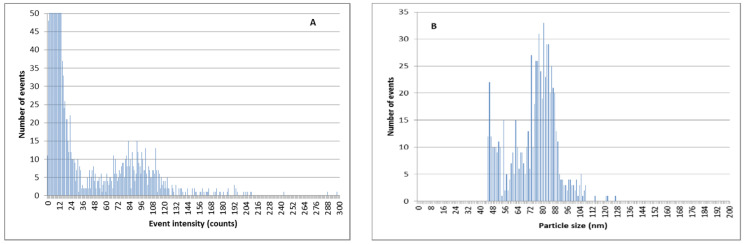
SP-ICPMS histograms of a mixture of Ag^+^ and PVP-AgNPs of 75 nm 5:1 after double extraction procedure with 50 mg resin. (**A**) Signal distribution. (**B**) Particle size distribution.

**Figure 11:**
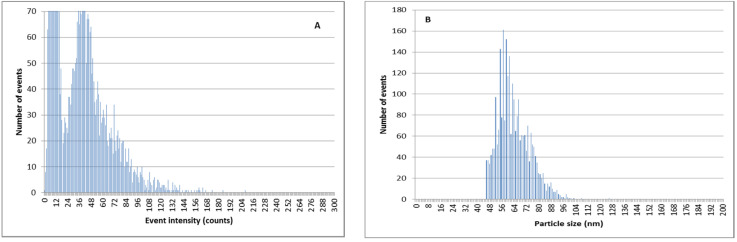
SP-ICPMS histograms of a mixture of Ag^+^ and citrate-stabilized AgNPs of 60 nm 5:1 after double extraction procedure with 50 mg resin. (**A**) Signal distribution. (**B**) Particle size distribution.

By using double ion-exchange pretreatment with the Dowex 50W-X8 prior to the SP-ICPMS analysis, the amount of ionic silver is reduced and the determination of AgNPs is remarkably better (Figure 10 and Figure 11). The results of the particle size determination in river water before and after the application of the ion-exchange pretreatment can be seen in Table 3. The improvement in the particle size determination, in comparison with the expected values, can be clearly observed.

## 4. Conclusions

In this paper, the effectiveness of the cation-exchange resin Dowex 50W-X8 for the separation of ionic silver from silver nanoparticles in natural water samples has been demonstrated. Although the extraction and separation is inhibited when cations such as Ca^2+^ are present, the addition of humic acids allows for the overcoming of this handicap because of its capacity to stabilize AgNPs [66]. The separation of both forms of silver can be carried out in either batch or column design. The latter allows the treatment of large quantities of samples. Under batch conditions, the resin could be reused up to four cycles. However, in column conditions, the resins could not be completely regenerated. Therefore, further investigation needs to be done regarding the regeneration method of the resin.

This separation methodology, when applied to river water samples, produced good results when a double extraction procedure was used, even for samples with ionic silver concentrations up to three times higher than that of the AgNPs. However, the usefulness of the method for wastewater samples could not be established. The high conductivity of the sample, which indicates higher amounts of ions in the sample, seems to influence the proper retention of silver ions. This problem has been previously observed for nickel [67]. Although the dilution of the sample could improve the results, AgNPs will also be diluted, and this could be a problem when the concentration is already very low. Therefore, additional research is needed with these types of waters in order to improve the results. The separation of Ag^+^ and AgNPs is also useful for determining the particle size distribution of silver nanoparticles present in river waters by SP-ICPMS when a high amount of ionic silver is present. The combination of the ion-exchange resin for extracting Ag^+^, with SP-ICPMS significantly improves the particle size determination for smaller nanoparticles (60 nm). This is due to the diminution of the silver ions signals that otherwise overlap with the silver nanoparticle signal. The discrimination between the continuous and noisy signals of the dissolved silver and AgNP signals are clearly better when the concentration of ionic silver decreases. This fact has previously been observed in a study of AgNP size and particle number concentration determination in soil aqueous leachates, carried out by Torrent et al. [62]. In that work, the authors proposed a cloud point extraction methodology to separate Ag^+^ and AgNPs. Although the results of both separation systems are similar, ion-exchange methodology is much simpler and easier to carry out.

## Figures and Tables

**Table 1:** Main chemical characteristics of river waters and wastewater used in this work.

	River Water 1	River Water 2	Wastewater
Conductivity (μS·cm^−1^)	314	367	956
pH	8.3	8.2	7.2
TOC (mg (C)·L^−1^)	1.975	1.815	-
Alkalinity (mg (CaCO_3_)·L^−1^)	107.3	117.1	-
Sodium (mg (Na^+^)·L^−1^)	22.88	22.77	115.96
Potassium (mg (K^+^)·L^−1^)	1.74	1.80	22.22
Magnesium (mg (Mg^2+^)·L^−1^)	5.81	6.67	12.87
Calcium (mg (Ca^2+^)·L^−1^)	28.99	34.77	74.48

**Table 2:** Instrumental characteristics and measurement conditions.

Agilent 5100 Vertical Dual View ICP-OES		
Instrumental characteristics	RF power	1200 W
	Pump speed	12 rpm
	Nebulizer chamber	Double pass glass cyclonic
	Nebulizer	Concentric glass
	Torch inner diameter	1.8 mm
	Nebulizer flow rate	0.7 L·min^−1^
	Argon gas flow rate	12 L·min^−1^
	Plasma configuration	Axial (double vision)
	Wavelength selector	Echelle polychromator
Data acquisition parameters	Ag wavelength	328.068 nm
	Detector	Charge-coupled device (CCD)
	Reading time	1 s
	Readings per replicate	3
**Agilent 7500c ICP-MS**		
Instrumental characteristics	RF power	1500 W
	Sample uptake rate	0.3 mL·min^−1^
	Nebulizer chamber	Double pass scott
	Nebulizer	Babington
	Torch inner diameter	2.5 mm
	Nebulizer gas flow rate	1.1 L·min^−1^
	Sampling cone	Ni, 1 mm aperture diameter
	Skimmer cone	Ni, 0.4 mm aperture diameter
	Argon gas flow rate	15 L·min^−1^
	Analyzer	Quadrupole
	Detector	Electron multiplier
Data acquisition parameters	Single particle measuring mode	
	Isotopes monitored	107
	Dwell time	10 ms
	Acquisition time	60 s
	Points per spectral peak	1
	Readings per replicate	5730

**Table 3:** Particle size of the AgNPs determined by SP-ICPMS in a mixture of Ag^+^:AgNPs 5:1 before and after a double extraction with Dowex 50W-X8 cation-exchange resin.

Manufacturer Value	Calculated Size before Extraction	Calculated Size after Double Extraction
79 nm	81 nm	78 nm
59 nm	74 nm	62 nm

## Data Availability

Data available upon request.
